# The Transcription Factor C/EBP-β Mediates Constitutive and LPS-Inducible Transcription of Murine SerpinB2

**DOI:** 10.1371/journal.pone.0057855

**Published:** 2013-03-05

**Authors:** Ekemini A. Udofa, Brett W. Stringer, Padmaja Gade, Donna Mahony, Marguerite S. Buzza, Dhananjaya V. Kalvakolanu, Toni M. Antalis

**Affiliations:** 1 Department of Physiology, Center for Vascular and Inflammatory Diseases, University of Maryland School of Medicine, Baltimore, Maryland, United States of America; 2 Queensland Institute of Medical Research, Herston, Queensland, Australia; 3 Department of Microbiology and Immunology, University of Maryland School of Medicine, Baltimore, Maryland, United States of America; 4 Greenebaum Cancer Center, University of Maryland School of Medicine, Baltimore, Maryland, United States of America; University of Crete, Greece

## Abstract

SerpinB2 or plasminogen activator inhibitor type 2 (PAI-2) is highly induced in macrophages in response to inflammatory stimuli and is linked to the modulation of innate immunity, macrophage survival, and inhibition of plasminogen activators. Lipopolysaccharide (LPS), a potent bacterial endotoxin, can induce SerpinB2 expression via the *toll*-like receptor 4 (TLR4) by ∼1000-fold over a period of 24 hrs in murine macrophages. To map the LPS-regulated *SerpinB2* promoter regions, we transfected reporter constructs driven by the ∼5 kb 5'-flanking region of the murine *SerpinB2* gene and several deletion mutants into murine macrophages. In addition, we compared the DNA sequence of the murine 5′ flanking sequence with the sequence of the human gene for homologous functional regulatory elements and identified several regulatory *cis*-acting elements in the human *SERPINB2* promoter conserved in the mouse. Mutation analyses revealed that a CCAAT enhancer binding (C/EBP) element, a cyclic AMP response element (CRE) and two activator protein 1 (AP-1) response elements in the murine *SerpinB2* proximal promoter are essential for optimal LPS-inducibility. Electrophoretic mobility shift (EMSA) and chromatin immunoprecipitation (ChIP) assays demonstrated that LPS induces the formation of C/EBP-β containing complexes with the *SerpinB2* promoter. Importantly, both constitutive and LPS-induced *SerpinB2* expression was severely abrogated in C/EBP-β-null mouse embryonic fibroblasts (MEFs) and primary C/EBP-β-deficient peritoneal macrophages. Together, these data provide new insight into C/EBP-β-dependent regulation of inflammation-associated SerpinB2 expression.

## Introduction

The inflammatory response is a double-edged sword. Properly orchestrated, it results in the clearing of foreign molecules and invading pathogens from the body. Uncontrolled, it may lead to organ damage, sepsis, and even cancer [1]–[3]. Many of the pathological manifestations of the inflammatory response are mediated by cytokines and other inducible gene products expressed by macrophages upon exposure to the gram-negative bacterial cell wall component LPS. As macrophages are key effectors of pathogen-induced innate immune responses, their survival is critical for initial pathogen neutralization and subsequent development of adaptive immune responses. One of the most LPS-inducible macrophage gene products known is the ovalbumin-like serine protease inhibitor (ov-serpin) SerpinB2, a widely recognized macrophage survival factor [4]; [5]. SerpinB2 was first identified as an inhibitor of urokinase-type plasminogen activator (uPA)[6]–[8], a serine protease involved in the degradation and turnover of the extracellular matrix through the activation of plasminogen [7]; [9]. Such function requires SerpinB2 to be secreted from the cell yet SerpinB2 exists primarily as a nonglycosylated intracellular protein [10]. Over the past decade, intracellular roles for SerpinB2 in cell survival [11]–[17], proliferation and differentiation [18]–[21], signal transduction [15]; [22]; [23] and innate immunity [24]–[28], have been described.

The *SerpinB2* gene is highly regulated in a cell type specific manner analogous to that of cytokines and oncogenes [29]; [30]. It is one of the most responsive genes known [31], and can be induced over 1000-fold by LPS [31]–[32], and is up-regulated by a range of inflammatory mediators [9]. LPS activates immune responses through multiple signalling pathways. The *toll*-like receptor 4 (TLR4) is responsible for the recognition of LPS and other microbial products and plays a central role in the initiation of innate immune responses, including cytokine release. The binding of LPS to TLR4 on the surface of macrophages leads to the recruitment of adaptor molecules and the activation of protein kinases, generating signals to the nuclear factor-κB (NF-κB), mitogen-activated protein kinase (MAPK) and/or phosphoinositide 3(PI3)-kinase pathways [33].

In studies aimed at identifying LPS-inducible pro-survival factors downstream of p38 MAPK, SerpinB2 was identified as a factor whose expression was upregulated by cooperation of the IKKβ/NF-κB and p38 MAPK/CREB pathways [16]. Our previously published data indicated that *SerpinB2* is distinctly regulated from other LPS-inducible genes in terms of kinetics, LPS dose response and sensitivity to IFN-γ co-stimulation [4]; however, the *cis*-acting elements in the *SerpinB2* promoter responsible for LPS-dependent transcription in macrophages and the specific LPS-responsive transcription factors that bind the *SerpinB2* promoter were not defined. Here we show that LPS induction of *SerpinB2* is dependent upon *cis*-acting regulatory sequences in the region between nucleotides −189 and −539 of the murine *SerpinB2* promoter, and is critically dependent upon a C/EBP binding site at −203/−195. C/EBP-β directly bound to this site *in vivo* and its deficiency abrogated constitutive *SerpinB2* expression and *SerpinB2* induction by LPS. Importantly, a C/EBP-β phospho-acceptor site was found to negatively regulate LPS-induced SerpinB2 promoter activity. Together, these findings provide new insight into the transcriptional regulation of the *SerpinB2* gene.

## Experimental Procedures

### Cell Culture

Murine macrophage RAW 264.7 cells (ATCC TIB-71) were maintained in RPMI 1640 media (Gibco BRL), supplemented with 2 mM L-glutamine (Gibco BRL), 10% serum supreme (BioWhittaker), 200 µg/ml penicillin, 100 µg/ml streptomycin, 25 mM N-2-hydroxyethylpiperazine-N-2-ethane sulphonic acid (HEPES) and 25 mM sodium bicarbonate, in 5% CO_2_ and 95% humidified air atmosphere at 37°C. Wild-type (*Cebpb^+/+^*) and knockout (*Cebpb^−/−^*) mouse embryonic fibroblasts (MEFs) [34] were grown in Dulbecco's modified Eagle's medium (Invitrogen) supplemented with 10% fetal bovine serum and 1% penicillin/streptomycin/glutamate solution (Cellgro). Primary peritoneal macrophages were obtained by injecting C57BL/6 mice with 3% thioglycollate broth followed by peritoneal lavage 3–5 days later, and maintained in 50% DMEM/F12 media (Gibco BRL). Precautions were taken to exclude bacterial lipopolysaccharide contamination from all cell cultures through the use of certified LPS-free serum. Cell viability was determined using the trypan blue (Sigma) dye exclusion method. All cultures were routinely checked to exclude Mycoplasma infection by nuclear staining using Hoechst stain 33258 (Sigma) and the MycoAlert Detection Kit (Lonza). Bacterial lipopolysaccharide (LPS) (*Salmonella minnesota* Strain Re595) was obtained from Sigma.

### Real-time Quantitative PCR (qPCR)

The RNeasy Mini Kit (Qiagen) was used to isolate total RNA from cells. For cDNA synthesis, 1 µg of total RNA was reverse transcribed using TaqMan® Reverse Transcription Reagents (Applied Biosystems). qPCR was performed using TaqMan® Gene Expression 20X primers for Serpinb2/SerpinB2 (Mm00440905_m1), Cebpb (Mm00843434_s1) and β-actin (Mm00607939_s1) (Applied Biosystems).

### DNA Sequence Analysis

The DNA sequence of the murine *SerpinB2* promoter was determined by sequencing the pUC-based plasmids pDB9406, pDB9402-41 and pDB9402-42, in addition to plasmids prepared from pDB9402-41 and pDB9402-42 containing deletions introduced by exonuclease III digestion. Plasmids pDB9406, pDB9402-41 and pDB9402-42 containing genomic DNA isolated from a λFIXII (Stratagene) genomic library prepared from a 129 mouse strain, were kindly provided by Dr. Dominique Belin, University of Geneva. pDB9406 contains a 4.4 kb *Eco*RI*/Spe*I genomic fragment spanning the transcription initiation site; pDB9402-41 and pDB9402-42 contain a 1.2 kb *Eco*RI genomic fragment, located immediately upstream of the pDB9406 *Eco*RI fragment, cloned in opposite orientations. Subcloned inserts were verified by restriction enzyme digestion and DNA sequencing. The nucleotide sequence of the 4480 bp murine *SerpinB2* gene 5′ flanking region was determined using the ABI PRISM dye terminator cycle sequencing ready reaction kit (Perkin-Elmer) and a PE 373A sequencer (Perkin-Elmer). This sequence was deposited in GenBank/EMBL/DDBJ Data Bank with Accession No. AF339731.

### Western Blot Assays

Whole cell lysates were prepared in RIPA buffer (10 mM Tris, 150 mM NaCl, 1%Triton X-100, 0.5% NP-40, 0.5% deoxycholate, 0.1% SDS), proteins were separated on 4–12% Bis-Tris NuPAGE gels (Invitrogen), and transferred to PVDF membranes. Membranes were subsequently blocked with 5% milk in PBS-T (1X PBS, 0.1% Tween-20), and incubated with primary antibodies overnight. Affinity purified rabbit anti-mouse SerpinB2 antibodies were prepared after immunization with a purified recombinant GST-murine SerpinB2 fusion protein produced in *E.* coli as in [35]. Other antibodies used for western blot assays include: C/EBP-β (sc-150) (Santa Cruz Biotechnologies), glyceraldehyde-3-phosphate dehydrogenase (GAPDH) and α/β tubulin (Cell Signaling Technology, Inc.).

### Construction of SerpinB2 Reporter Gene Plasmids

The PCR primers DW5’LUC, containing a *Kpn* I restriction site, and DW3’LUC, containing an *Xho* I restriction site, were used to PCR amplify and clone the *SerpinB2* promoter (−3261 to +92) from pDB9406 into the *Kpn* I/*Xho* I polylinker restriction sites of pGL3 Basic (Promega) to produce pGLmP-3261. The *Eco*R I insert of pDB9402-42 was sub-cloned immediately upstream of the *SerpinB2* promoter *Eco*R I site (−3261) of pGLmP-3261 to produce pGLmP-4480. Additional murine *SerpinB2* luciferase reporter constructs (pGLmP-2751, pGLmP-2614, pGLmP-1686, pGLmP-1341, pGLmP-694, pGLmP-539 and pGLmP-189) were generated by digesting pGLmP-3261 with *Eco*R I and a second restriction enzyme (*Sfi* I, *Apa* I, *Bst*X I, *Bsu*36 I, *Pac* I, *Hae* III and *Apo* I respectively), blunt ending the resultant 5′ or 3′ overhangs with T4 DNA polymerase (NEB) and re-ligating the vector ends with T4 DNA ligase. The PCR primers BSPCR2 and DW3’LUC were used to subclone the murine *SerpinB2* promoter regions −87 to +92 into the *Kpn* I/*Xho* I polylinker restriction sites of pGL3 Basic to produce pGLmP-87. The control empty vector was pGL3 Basic (Promega).

Luciferase reporter constructs containing mutations in the *SerpinB2* promoter LPS-responsive regions E box, PU.1, Oct-1 and C/EBP (pGLmP-539mEbox, pGLmP-539mPU.1, pGLmP-539mOct and pGLmP-539mC/EBP respectively) were generated as described [36]. The mutant oligonucleotide PCR primer sequences are provided in Fig. S1, and were used as follows: *pGLmP-539mEbox:* mPAI2mEboxa and mPAI2mEboxb; pGLmP*-539mPU.1:* mPAI2mPU.1a and mPAI2mPU.1b; pGLmP*-539mOct:* mPAI2mOct-2a and mPAI2mOct-2b; *pGLmP-539mC/EBP:* mPAI2mCEBPa and mPAI2mCEBPb. The oligonucleotide PCR primers used to generate mutants, pGLmP-539mCRE, pGLmP-539mAP-1a, and pGLmP-539mAP-1b were reported previously [37]. The flanking oligonucleotide PCR primers were RVprimer3 (Promega) and GLprimer2 (Promega). pGLmP-539 was used as the template in each case. Recombinant PCR products were digested with *Eco*R I and *Xho* I and cloned between the *Eco*R I and *Xho* I restriction sites of pGLmP-539 in place of the −539/+92 region of the wild-type murine *SerpinB2* promoter. Sequence verified constructs were used in the experiments.

### Transient Transfection and Luciferase Assays (RAW264.7 Macrophages)

RAW 264.7 cells (2×10^7^) growing in log phase were transfected with the indicated luciferase reporter plasmid (20 µg) along with the pRL-thymidine kinase (TK) (Promega) internal control reporter plasmid (2 µg) by electroporation using a Bio-Rad Gene Pulser with a Capacitance Extender (0.25 kV, 960 µFd). pGL3 control plasmid which encodes the SV40 promoter and enhancer was included as a positive control for transfection efficiency, and as an internal standard for promoter and enhancer activities. Transfected cells were transferred to 10 ml of pre-warmed media in 6-well tissue culture plates, divided into two identical cell pools and incubated 16 hrs in a 5% CO_2_ and 95% humidified air atmosphere at 37°C either in the presence or absence of 100 ng/ml LPS. Luciferase activity was measured using a Dual-Luciferase Reporter Assay System kit (Promega). Measurements represent the results of at least three independent experiments. Promoter activity is expressed as the number of firefly luciferase light units normalized either to pRL-TK renilla luciferase light units or to cellular protein concentration (where co-transfected C/EBP-β or LPS affected pRL-TK activity). Protein concentration was determined using the Bio-Rad protein microassay reagent.

### Lentiviral shRNAs, Packaging and Transduction

pLKO.1-puro lentiviral vectors carrying short hairpin RNAs (shRNA) specific for human and mouse *cebpb* were used in these studies. Because the human and mouse *cebpb* 3′ untranslated regions are not identical, these species-specific shRNAs cannot knockdown expression of endogenous C/EBP-β when used on cells of the other species; therefore we used the human *CEBPB* shRNA as a control in these experiments as in [38]. To produce lentiviral particles, HEK-293T cells were transfected with a mixture of plasmids: each shRNA expression plasmid (1 µg), pCMV-ΔR8.2dvpr packaging plasmid (0.75 µg), and pCMV-VSV-G envelope plasmid (0.25 µg) using Lipofectamine 2000 reagent (Invitrogen). The lentiviral supernatant was collected 48 hrs after transfection, cleared by centrifugation at 2,000 g for 10 mins and passed through a 0.45 µm filter. The target cells were treated with the lentiviral supernatant and 8 µg/ml Polybrene (American Bioanalytical) for 24 hrs. The lentiviral supernatant was replaced with fresh growth media and incubated further for 72 hrs to allow for effective gene knockdown. C/EBP-β knockdown was confirmed by western blot analysis.

### Transient Transfection and Luciferase Assays (MEF Cells)

*Cebpb* ^−/−^ MEFs (1×10^5^) [34] were transfected with the indicated luciferase reporter plasmid (400 ng) along with a β-actin-β-galactosidase reporter plasmid (200 ng) by electroporation using the Invitrogen Neon™ system (1 pulse, 1350 V, 30 msec). Transfected cells were transferred to pre-warmed media in 24 well plates and incubated for 48 hrs prior to incubation with LPS (100 ng/ml) for 4 hrs where indicated. In some experiments, plasmids encoding C/EBP-β or C/EBP-β phospho-acceptor mutants (T^188^A, T^217^A, S^64^A) [38]–[40] or control vector were co-transfected (0.6–1.0 µg total DNA). Luciferase activity was determined and normalized to that of β-galactosidase [38] using the Luciferase Assay System and β-Galactosidase Enzyme Assay System Kits, respectively (Promega). Each experiment was repeated at least three times, and triplicate samples were employed for each sample. Expression of the C/EBP-β phospho-acceptor mutant proteins was checked for equal expression by western blot.

### Electrophoretic Mobility Shift Assays

Radiolabelled, double-stranded oligonucleotide probes for gel shift assays were prepared using T4 polynucleotide kinase and [γ-^32^P]-ATP. RAW 264.7 nuclear extracts were prepared by the detergent lysis method [41]. DNA binding reactions (25 µl) containing 10 mM HEPES pH 7.9, 10 µg/ml BSA, 2 mM DTT, 30% glycerol, 20% Ficoll-400, 1 µg poly (dI-dC), 6 µg of nuclear extracts and 10,000 to 20,000 cpm (0.05 to 0.2 ng) of radiolabelled probe were performed for 20 minutes at room temperature. Binding reaction products were resolved by electrophoresis at room temperature on 5% polyacrylamide gels (29∶1 acrylamide:bis-acrylamide (Bio-Rad)) in 1x TBE at 10V/cm. For supershift assays, nuclear extracts were pre-incubated 2 hrs on ice with 4 µg of specific C/EBP antibody. Antibodies were purchased from Santa Cruz: anti-C/EBP-α (sc-61), anti-C/EBP-β (sc-150), anti-C/EBP-δ (sc-151) and anti-C/EBP-ε (sc-158).

### Chromatin Immunoprecipitation (ChIP) Assay

ChIP assays were performed using a commercially available Magna-ChIP™ kit (Millipore), as recommended by the manufacturer, with minor modifications. Briefly, after crosslinking the chromatin with 1% formaldehyde at room temperature for 10 min and neutralizing with glycine for 5 min at room temperature, cells were washed with cold PBS, scraped and collected on ice. Cells extracts were prepared using a commercially available kit (Millipore). Nuclear lysates were sonicated 5 times for 15 sec with 1 min intervals on ice using a Sonic Dismembrator (Fisher). An equal amount of chromatin was immunoprecipitated at 4°C overnight with at least 1 µg of the following antibodies: C/EBP-β (sc-150X), p-C/EBP-β (T217) (sc-16993X), normal rabbit IgG (sc-2027)(Santa Cruz Biotechnologies) and RNA polymerase II (Clone CTD4H8)(Millipore). Immunoprecipitated products were collected after incubation with Protein G coated magnetic beads (Millipore). The beads were washed, the bound chromatin was eluted in ChIP Elution Buffer (Millipore) and the proteins were digested with Proteinase K for 2 hrs at 62°C. The DNA was then purified using the QIAquick PCR Purification Kit (Qiagen). DNA was amplified by semi-quantitative PCR or by qPCR using the SYBR green method and primers specific for the *SerpinB2* proximal promoter: forward (−338/−315) ^5′^AAGACTCCCACAGATGGTGGCTGT^3’^; reverse (−5/+19) ^5′^TTCTTGGAAAGCTGGCACTGTGTG^3’^.

### Statistical Analysis

Data are presented as mean ± SEM per group. Results were analyzed using the analysis of variance (ANOVA) or Student’s t test where relevant. P-values <0.05 were considered significant.

## Results

### The SerpinB2 Gene is Highly Responsive to LPS

**Figure 1:**
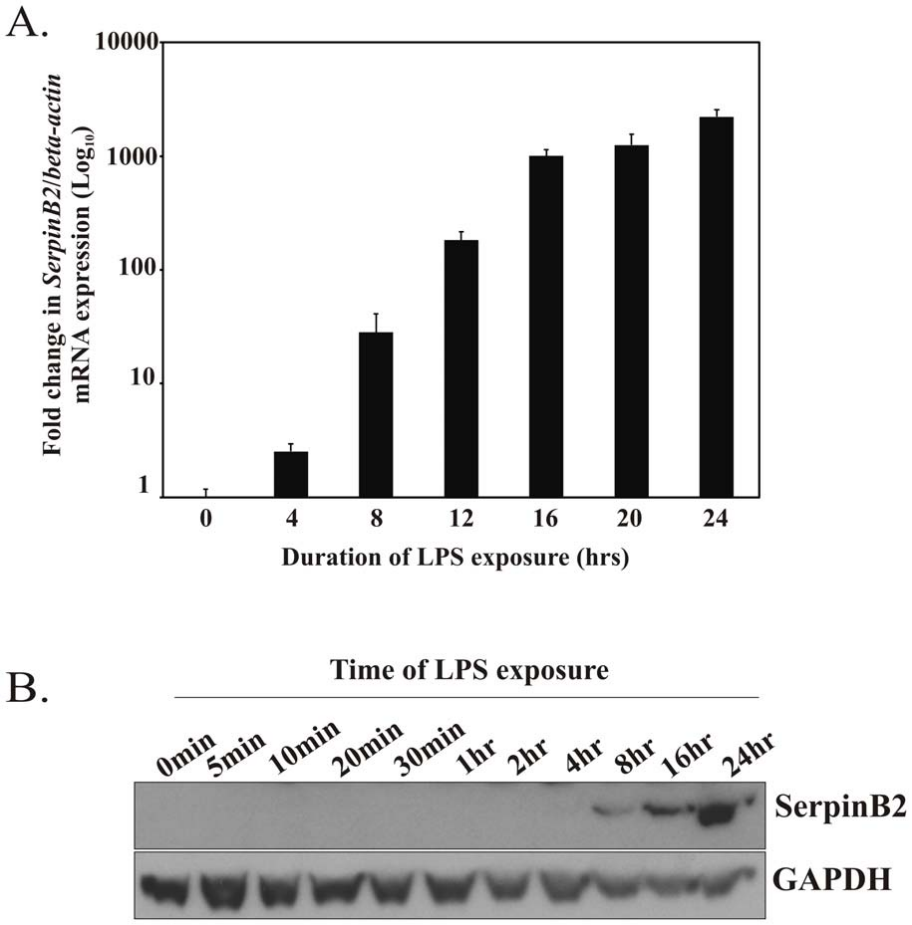
LPS induction of *SerpinB2* mRNA and protein expression in murine macrophage cells. RAW264.7 macrophages were treated for the indicated times with 100 ng/ml LPS. *(A)* qPCR analysis of murine *SerpinB2* mRNA levels, relative to β-actin. The plot is representative of at least two independent experiments performed in triplicate. *(B)* Immunoblot analysis of SerpinB2 protein expression in whole cell lysates. Blot was reprobed for GAPDH as a loading control.

When RAW264.7 macrophages were exposed to LPS, *SerpinB2* mRNA was detectable as early as 30 min following LPS challenge, reaching maximal levels at 24 hrs (Fig. 1A). A similar strong induction of LPS-inducible *SerpinB2* mRNA expression has been reported previously in murine peritoneal macrophages and human peripheral blood mononuclear cells [4]; [32]. LPS-induced *SerpinB2* expression involves both an increase in gene transcription and stabilization of the mRNA [5]; [29]; [42]–[44]. SerpinB2 protein expression was also induced as has been reported in other cell types [31], and detectable after 8 hrs of LPS treatment (Fig. 1).

### The SerpinB2 Proximal Promoter Confers LPS Responsiveness

**Figure 2:**
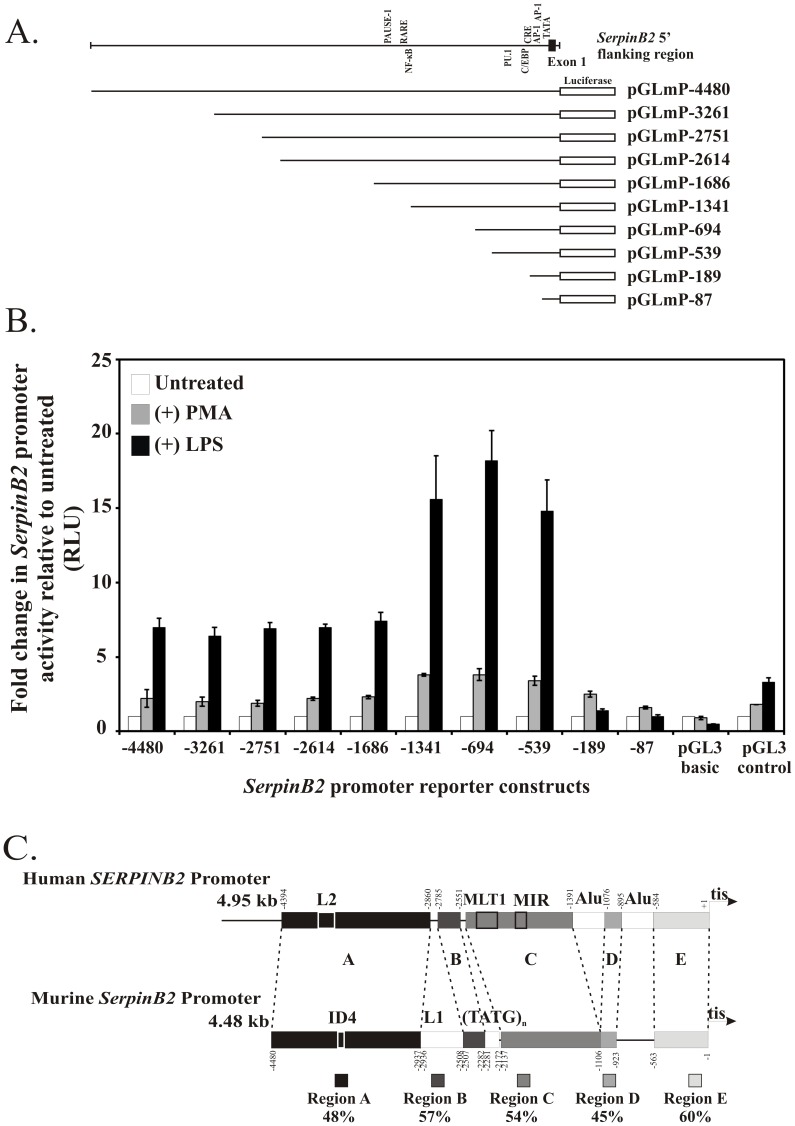
Deletion reporter gene analysis of LPS and PMA-responsive regions in the murine *SerpinB2* promoter. *(A)* Schematic representation of the murine *SerpinB2* promoter and 5′ deletion luciferase reporter constructs. The murine 5′ flanking region from −4480 to +92 was inserted upstream of the luciferase reporter gene and 5′ deletions were generated using restriction enzyme sites or with specific oligonucleotide PCR primers. Construct names indicate the most 5′ nucleotide of murine *SerpinB2* 5′ flanking sequence. *(B)* RAW 264.7 macrophages were transiently transfected with the indicated murine *SerpinB2* promoter-luciferase reporter constructs and control plasmids. Cells were either left untreated or treated with 100 ng/ml LPS or 40 ng/ml PMA for 16 hrs. Shown is the relative luciferase reporter gene activity following treatment. The results represent the mean and SEM of four independent experiments. *(C)* DNA sequence conservation between the human and murine SerpinB2 5′ flanking regions. Schematic representation of the human and murine *SerpinB2* 5′ flanking regions with regions of nucleotide sequence identity indicated by the same colored boxes. Homologous regions are interrupted by repetitive sequence elements in both 5′ flanking regions. Alu = Alu repeat, ID4 =  ID4 short interspersed nuclear repeat (SINE), L1 =  L1 long interspersed nuclear element (LINE), L2 =  L2 LINE, MIR = MIR SINE, MLT1L = MLT1L long terminal repeat (LTR), (TATG)_n_ = TATG tetranucleotide repeat, tis = transcription initiation site.

To investigate *cis*-acting regulatory elements responsive to LPS in the 5′ flanking region of the murine *SerpinB2* gene, nucleotides −4480 to +92 and a series of generated deletion mutants of the 5′ flanking region were cloned upstream of a promoter-less firefly luciferase reporter gene (pGL3 Basic) (Fig. 2A). The reporter constructs were then transiently transfected into sub-confluent RAW264.7 macrophages and assayed for luciferase activity in the presence and absence of LPS or PMA, for comparison. PMA-induced *SerpinB2* gene regulation has been extensively studied in human macrophage cell lines [45]–[47], and has been shown to occur through several proximal and distal AP-1 responsive elements [30]; [37]; [47]–[51]. As shown in Fig. 2B, the *SerpinB2* 5′ flanking region from −4480 to +92 directs both PMA- and LPS-inducible transcription, approximately 2-fold and 7-fold, respectively. Deletion of the murine *SerpinB2* promoter from −1686 to −1341 increased LPS-inducibility to approximately 16-fold, indicating the presence of a silencer element in this region. Further deletion beyond −539 abolished the LPS-response of the promoter, indicating the presence of an essential LPS response element in the region between −539 and −189; however, the response of the murine *SerpinB2* promoter to PMA is less affected by this deletion. While deletion of the *SerpinB2* promoter from −189 to −87 eliminated the LPS response and marginally reduced the PMA response, the −87 murine *SerpinB2* promoter construct was still partially responsive to PMA, indicating that *cis*-acting elements mediating the response of the murine *SerpinB2* promoter to PMA also lie downstream of nucleotide −87.

### Sequence Conservation within the Human and Murine SerpinB2 Proximal Promoters

**Figure 3:**
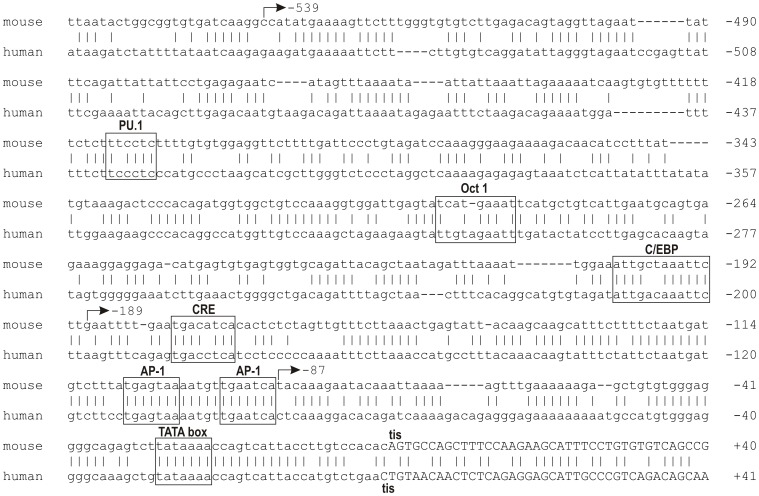
Potential cis-acting regulatory elements in the LPS responsive region of the murine *SerpinB2* promoter −**563/−1.** Mouse and human nucleotide sequences were aligned using Clustal W software. *Cis*-acting elements conserved between the human and murine SerpinB2 promoters are boxed and labeled. AP-1 =  activator protein 1; C/EBP =  CCAAT enhancer binding protein; CRE = cAMP response element; Oct1 =  octamer transcription factor 1/POU2F1; PU.1 =  purine box binding protein 1. The putative transcription initiation site (tis) is indicated and exon 1 is presented in uppercase. The location of the 5' ends of the −539, −189 and −87 reporter constructs are also shown.

To look for potential *cis*-acting elements that might mediate transcription of the murine *SerpinB2* gene and the response to LPS, we aligned the murine and human *SerpinB2* 5′ flanking regions. We reasoned that the presence of evolutionarily conserved, potential transcription factor binding sites in this region might play a role in the regulation of *SerpinB2* gene expression [52]. The presence of several repetitive sequence elements delineated five broadly homologous regions (A-E) between the human and murine promoters (Fig. 2C). The proximal promoter (Region E), which contains the essential LPS response element, exhibited the greatest homology. Further analysis of Region E revealed that several of the *cis*-acting regulatory elements defined in the human *SerpinB2* proximal promoter are conserved in the murine *SerpinB2* promoter (Fig. 3). Specifically, a TATA consensus sequence is located 23 to 29bp upstream from the transcription initiation site, similar to the position of the TATA box of the human *SERPINB2* gene [53]; [54]. A putative CCAAT enhancer binding protein (C/EBP) site is present at −192/−203, two potential activator protein 1 (AP-1) binding sites are found at nucleotides −88/−94 and −100/−106, and a putative cyclic AMP response element (CRE) is present at −172/−177. Both of the AP-1 sites are identical in sequence to those identified in the human *SERPINB2* promoter, and the CRE differs in the identity of a single central nucleotide [37]; [55]. A consensus E box (−538/−533), as well as potential binding sites for PU.1 (−412/−407) and Oct-1 (−296/−288) were also identified within the proximal promoter region (Fig. S2). Further upstream, a retinoic acid response element at −1349/−1340, a PAUSE-1 silencer element at −1540/−1528 and a NF-κB p65 binding site at −1342/−1351 (Fig. S2) are also well conserved [16]; [30]; [56].

### A C/EBP, CRE and Two AP-1 Sites in the Murine SerpinB2 Gene Proximal Promoter are Essential for Optimal LPS-inducible Transcription

**Figure 4:**
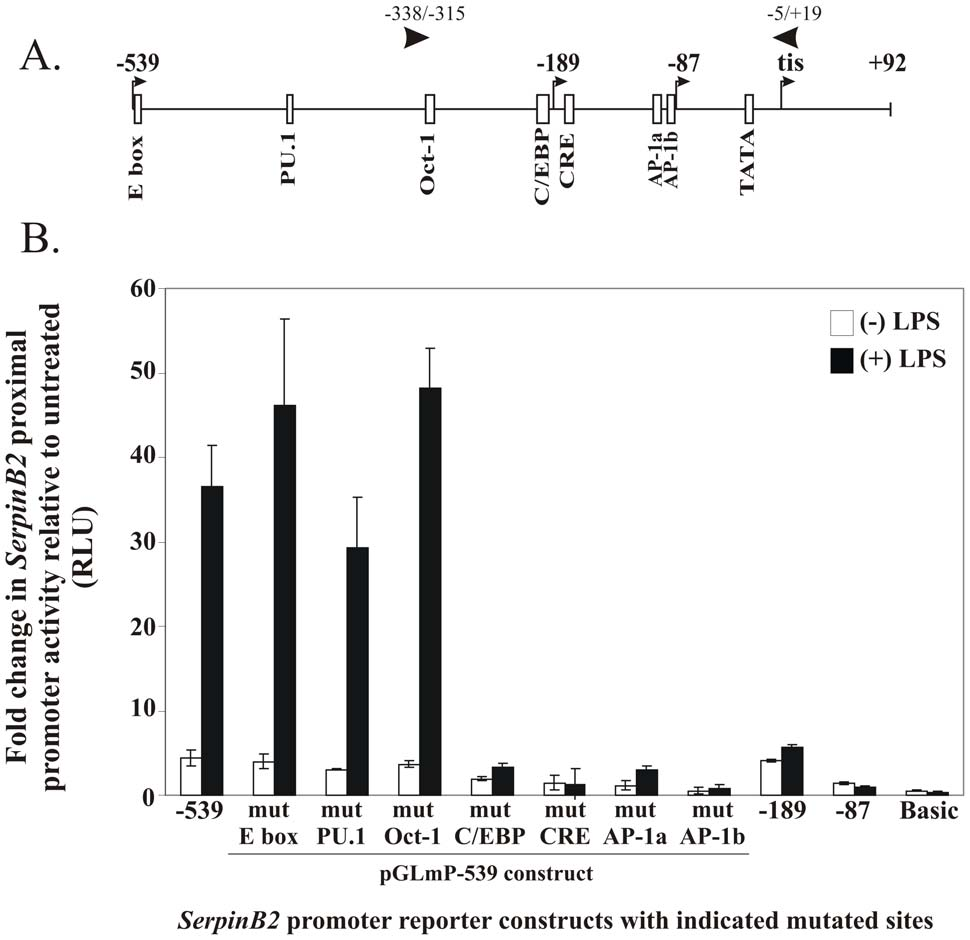
Identification of *cis*-acting elements required for the LPS-responsiveness of the murine *SerpinB2* promoter. *(A)* Schematic representation of the −539/+92 region of the murine *SerpinB2* promoter with the location of candidate *cis*-acting regulatory elements indicated with *boxes*. The location of the 5′ ends of the −539, −189 and −87 reporter constructs is also shown. Positions of murine *SerpinB2* proximal promoter-specific primers, 338/−315 and −5/+19 used in ChIP assays are indicated. *(B)* RAW 264.7 macrophages were transiently transfected with the indicated murine *SerpinB2* promoter-luciferase reporter constructs and either left untreated or treated with 100 ng/ml LPS for 16 hrs. The results show relative luciferase activity following LPS treatment and represent the mean and SEM of 4–7 independent experiments.

To investigate regulatory elements between −539 and −189 essential for LPS-inducible transcription, several candidate binding sites for transcription factors previously reported to mediate LPS-inducible transcription in other genes [57] were targeted by nucleotide substitution designed to disrupt transcription factor binding to the pGLmP-539 murine *SerpinB2* promoter-luciferase reporter gene construct (Fig. 4A). As shown in Fig. 4B, mutation of the consensus E box (−538/−533), PU.1 (−412/−407), or the variant Oct-1 (−296/−288) site did not decrease LPS-induced promoter activity. In contrast, mutation of the C/EBP site (−203/−192) completely eliminated promoter activity, similar to the levels observed for the −189 *SerpinB2* promoter deletion construct. Others have also recently implicated this C/EBP site in LPS-induced activation of the *SerpinB2* gene [58].

Considering the conserved sequence and position of the CRE and two AP-1 sites in the murine *SerpinB2* promoter and their demonstrated involvement in the PMA-responsiveness of the human *SERPINB2* promoter [37], these sites were also mutated by nucleotide substitution to investigate whether they played a role in the LPS response of the murine *SerpinB2* promoter. As shown in Fig. 4B, mutation of the CRE at −177/−172 or either of the two AP-1 sites at −106/−100 and −94/−88, completely or significantly reduced LPS-inducible luciferase activity from the murine *SerpinB2* promoter. These data show that the C/EBP element, as well as the CRE and both AP-1 *cis*-acting elements are critical for LPS-inducible transcription from the *SerpinB2* proximal promoter.

### The C/EBP Element is Bound by an LPS-induced Nuclear Factor from RAW 264.7 Macrophages

**Figure 5:**
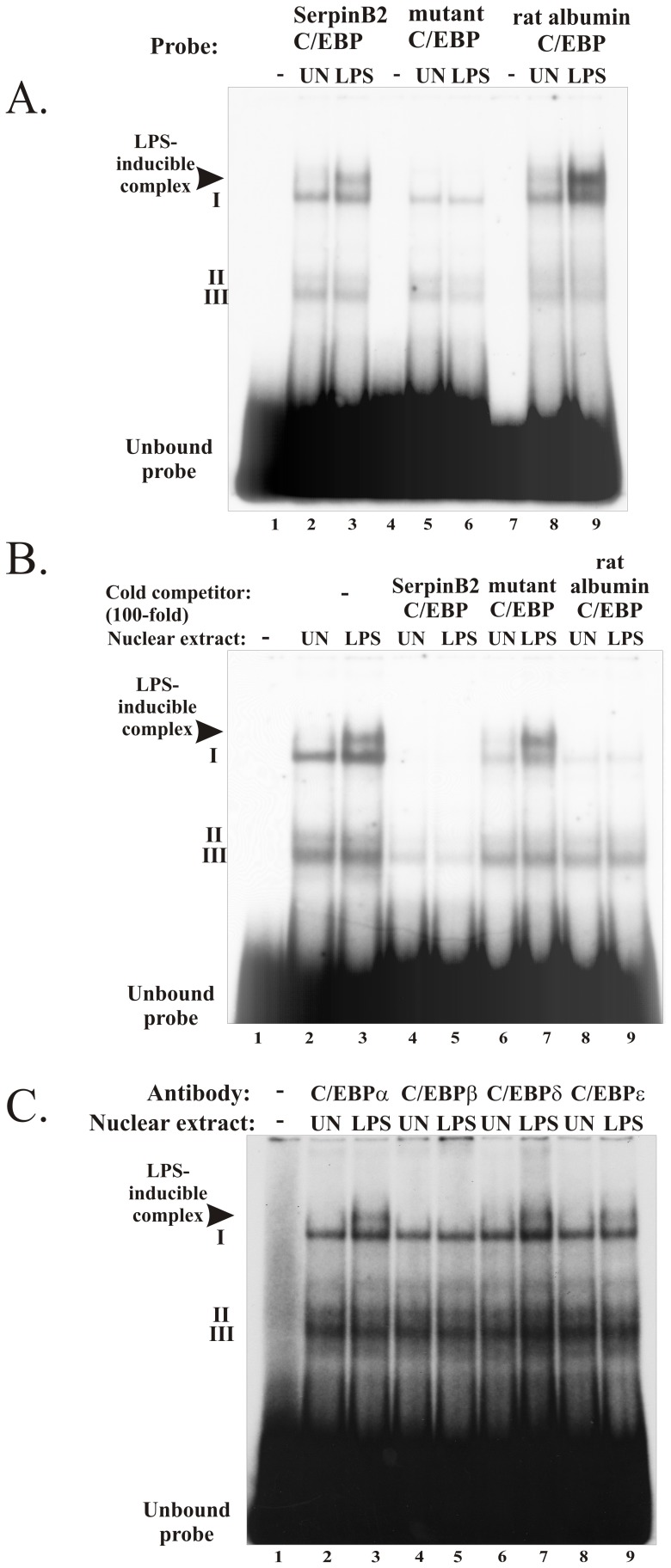
The LPS-inducible nuclear factor binding the murine *SerpinB2* proximal promoter C/EBP site contains C/EBP-β. *(A)* EMSA with nuclear extracts from untreated (UN) and 10 hr LPS treated (LPS) RAW 264.7 cells incubated with either the murine *SerpinB2* promoter −212/−185 probe containing an intact (SerpinB2 C/EBP) or mutated (mutant C/EBP) C/EBP site or with the rat albumin promoter DEI region C/EBP (rat albumin C/EBP) probe. *(B)* Cold competition EMSAs performed with the radiolabelled murine SerpinB2 promoter −212/−185 probe and a 100-fold molar excess of each of the double stranded oligonucleotides described in (*A*) demonstrate the specificity of the DNA-protein complexes. *(C)* Supershift assays were performed with the −212/−185 probe by after preincubation with the indicated specific C/EBP antibody. The LPS-inducible complex is indicated with an *arrowhead*.

Nuclear factor binding to the putative C/EBP element (−203/−192) was investigated by electrophoretic mobility shift assay (EMSA) using nuclear extracts from untreated and LPS-treated RAW 264.7 macrophages. Three different double stranded oligonucleotide probes were used for EMSA, representing (1) the putative *SerpinB2* C/EBP element (−203/−192), (2) a mutant *SerpinB2* element containing the same mutation as in pGLmP-539mC/EBP and (3) the rat albumin promoter distal element 1 (DEI) region containing a high affinity C/EBP binding site. Four bands of different mobilities, representing DNA-nuclear protein complexes were detected (Fig. 5A). Three of these bands (I, II, III) represent complexes with single stranded DNA (Fig. 5A) while the uppermost (slowest migrating) complex was induced by LPS. The LPS-inducible complex was not detected using the mutant C/EBP oligonucleotide probe and could be abolished by an excess of unlabeled double-stranded oligonucleotide, carrying either the same sequence (Fig. 5B, *lanes* 4 and 5) or the sequence of a known C/EBP binding site from the rat albumin promoter (Fig. 5B, lanes 8 and 9), but not by the mutated oligonucleotide (Fig. 5B, lanes 6 and 7). Together these data indicate that the putative *SerpinB2* C/EBP site at −203/−192 binds a LPS-inducible complex that is likely to contain a member of the C/EBP family of transcription factors.

### C/EBP-β is a LPS-induced Nuclear Factor that Binds to the C/EBP Element of the SerpinB2 Proximal Promoter

The C/EBP family of basic leucine zipper transcription factors are known for their roles in cellular differentiation and inflammation [59]. Consisting of six members, the C/EBP transcription factors can homo−/heterodimerize and display similar DNA binding specificities [60]. Four family members, C/EBP-α, C/EBP-β, C/EBP-δ and C/EBP-ε, are present in myeloid cells and play different roles in differentiating myeloid cells depending on the extracellular environment [61]. To determine which C/EBP proteins were involved in the formation of the different nucleo-protein complexes, and particularly of the LPS-inducible complex, EMSA was performed after incubating the nucleo-protein complexes with antibodies specific for C/EBP-α, C/EBP-β, C/EBP-δ and C/EBP-ε. Each antibody detects the carboxy-terminal DNA-binding region of the respective protein, so that pre-incubation of antibody with nuclear extract is expected to abolish DNA binding by EMSA [62]. Only antibodies against C/EBP-β abolished the formation of the LPS-inducible complex (Fig. 5C). Taken together these data show that the LPS-induced complex with the C/EBP element (−203/−192) contains C/EBP-β.

### C/EBP-β Mediates both Constitutive and LPS-induced SerpinB2 mRNA Expression in MEFs and Inflammatory Primary Macrophages

**Figure 6:**
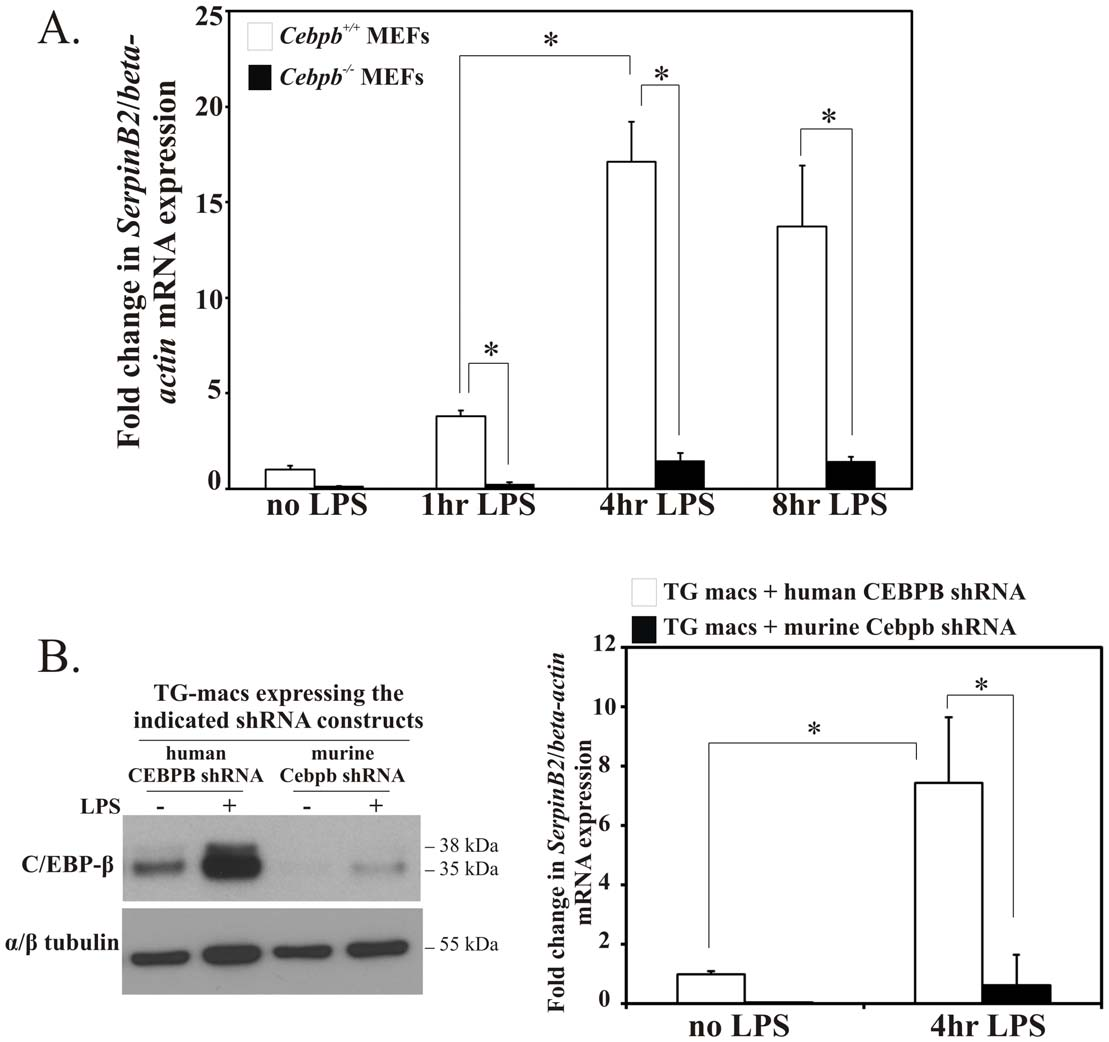
C/EBP-β is essential for constitutive and LPS-induced *SerpinB2* mRNA expression. *(A)* Endogenous *SerpinB2* mRNA expression is abrogated in *Cebpb^−/−^* MEFs compared to *Cebpb^+/+^* MEFs in the absence and presence of LPS. qPCR analysis of murine *SerpinB2* mRNA expression in untreated *Cebpb^+/+^* and *Cebpb^−/−^* MEFs, and after simulation with LPS (100 ng/ml) for the indicated times. *(B)* Endogenous SerpinB2 expression is abrogated in C/EBP-β-deficient inflammatory macrophages. Thioglycollate-elicited peritoneal macrophages (TG macs) were infected with human and murine specific lentiviral shRNAs. Human CEBPB shRNA serves as the non-silencing control since it does not target the murine Cebpb sequence [38]. Lentiviral transduced macrophages were stimulated with LPS (100 ng/ml) for 4 hrs. *Left:* Western blot analysis shows effective knockdown of endogenous C/EBP-β following infection with murine Cebpb shRNA and not human CEBPB shRNA. *Right:* qPCR analysis of murine SerpinB2 mRNA expression in the lentiviral transduced peritoneal macrophages. The results represent the mean and SEM of two independent experiments performed in duplicate or triplicate. (*, p<0.05, two-way ANOVA).

To investigate the importance of C/EBP-β to endogenous *SerpinB2* mRNA expression in response to LPS, we utilized C/EBP-β-null (*Cebpb* ^−/−^) and wild-type MEFs (*Cebpb^+/+^*), since RAW264.7 cells constitutively express endogenous C/EBP-β. Wild-type MEFs express low levels of endogenous *SerpinB2* and the absence of C/EBP-β attenuated endogenous *SerpinB2* mRNA expression. LPS stimulated an increase in *SerpinB2* mRNA expression in wild-type MEFs (Fig. 6A), whereas LPS-stimulated *SerpinB2* mRNA expression was significantly dampened in *Cebpb^−/−^* MEFs as compared to wild-type. We next tested if similar effects could be seen in thioglycollate-elicited inflammatory macrophages in which C/EBP-β expression was knocked down using species-specific lentiviral shRNAs (Fig. 6B, left). As was observed in MEFs, both constitutive and LPS-induced *SerpinB2* mRNA expression was significantly decreased in C/EBP-β-deficient inflammatory macrophages (Fig. 6B, right). These data show that C/EBP-β is critical for mediating constitutive and LPS-inducible transcription of endogenous *SerpinB2* mRNA.

### C/EBP-β Binds the Murine SerpinB2 Proximal Promoter in vivo in an LPS-inducible Manner

**Figure 7:**
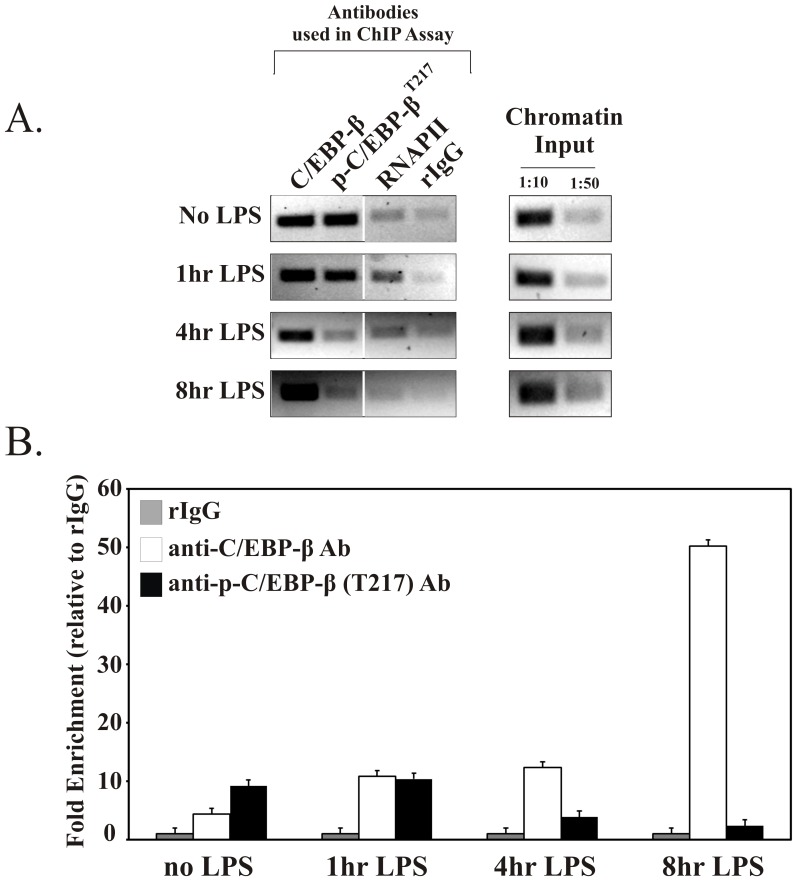
Differential recruitment of C/EBP-β and C/EBP-β^T217^ to the murine *SerpinB2* promoter *in vivo*. Transcription factor occupancy on the *SerpinB2* proximal promoter *in vivo* was determined by chromatin immunoprecipitation (ChIP) assay. RAW264.7 macrophages were treated in the presence or absence of LPS for the indicated times and chromatin immunoprecipitation performed using antibodies against C/EBP-β or p-C/EBP-β (T217). Soluble chromatin (600–700 ng) was immunoprecipitated with antibodies against C/EBP-β, p-C/EBP-β (T217), RNA Polymerase II (RNAPII), or a rabbit IgG (rIgG) control. *(A)* Typical PCR pattern obtained in ChIP assays using murine *SerpinB2* proximal promoter-specific primers, 338/−315 and −5/+19, as diagrammed in *top*. Recruitment of RNAPII to the *SerpinB2* promoter suggests active transcription following LPS stimulation. Minimal background was detected using the rabbit IgG control, indicative of the specificity of the ChIP reaction. *(B)* qPCR analysis of chromatin immunoprecipitated with antibodies against C/EBP-β, p-C/EBP-β (T217), and the rIgG control. The data are represented relative to rIgG signal using the 2^-ΔΔCt^ method.

We investigated the temporal dynamics of C/EBP-β recruitment to the murine *SerpinB2* promoter in response to LPS *in vivo* by chromatin immunoprecipitation (ChIP). RAW 264.7 cells were stimulated with LPS for up to 8 hrs, soluble chromatin was immunoprecipitated with antibodies against DNA binding proteins, and the enriched DNA amplified by both semi-quantitative and qPCR using *SerpinB2* proximal promoter specific primers (illustrated in fig. 4A). The results showed that C/EBP-β is constitutively present at the *SerpinB2* promoter as demonstrated by its association with the promoter in unstimulated macrophages and increased temporally in response to LPS reaching as much as 10-fold over unstimulated cells after 8 hrs (Fig. 7). Since changes in C/EBP-β phosphorylation states can affect C/EBP-β’s ability to transactivate target genes [38]; [63]; [64], we investigated recruitment to the *SerpinB2* proximal promoter of the T^217^ phosphorylated C/EBP-β isoform (p-C/EBP-β^T217^), which has been associated with cell survival [65]. p-C/EBP-β^T217^ was constitutively bound to the *SerpinB2* proximal promoter and also present after 1 hr of LPS stimulation (Fig. 7). In contrast to total C/EBP-β, the binding affinity of p-C/EBP-β^T217^ for the *SerpinB2* proximal promoter diminished with LPS stimulation at later timepoints (4 and 8 hrs)(Fig. 7). These data show an inverse relationship between C/EBP-β and p-C/EBP-β^T217^ recruitment, and indicate that T^217^-phosphorylated C/EBP-β may not be responsible for increased transcription from the *SerpinB2* promoter in response to LPS.

### C/EBP-β Promotes LPS-inducible Murine SerpinB2 Proximal Promoter Activity

**Figure 8:**
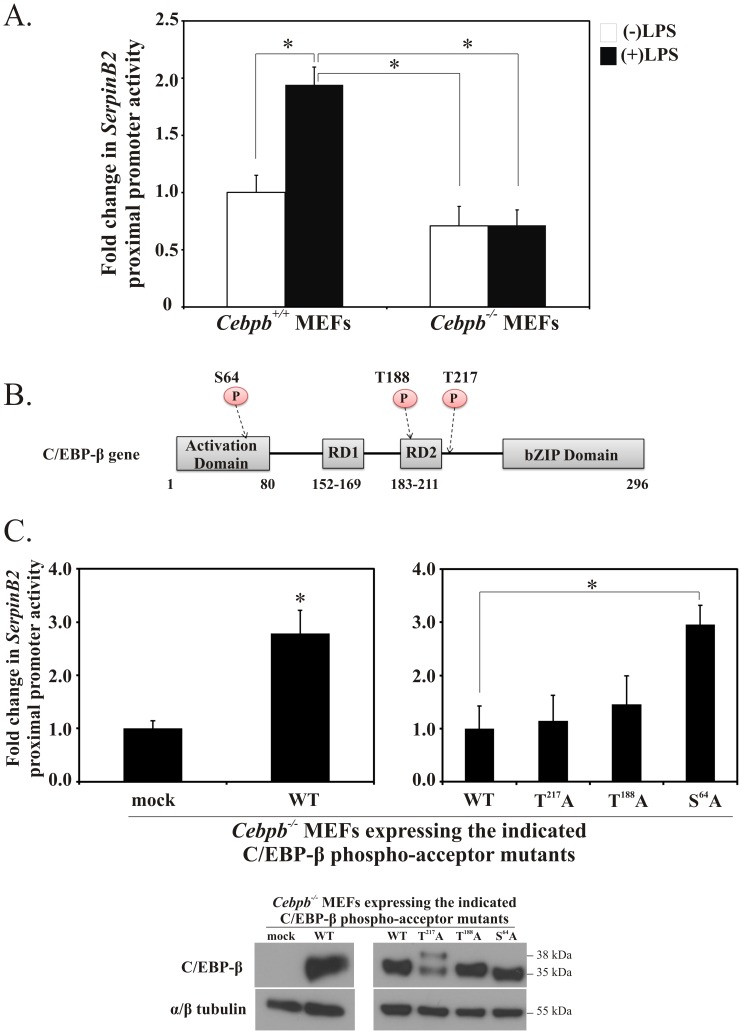
C/EBP-β is necessary for *SerpinB2* proximal promoter activity. *(A)* The *SerpinB2* proximal promoter is significantly activated in LPS-stimulated *Cebpb^+/+^* MEFs and not in *Cebpb^−/−^* MEFs. Murine *SerpinB2* gene promoter activity measured in *Cebpb^+/+^* and *Cebpb^−/−^* MEFs expressing the pGLmP-539 *SerpinB2* promoter-luciferase reporter in the presence or absence of LPS (100 ng/mL). Cells were co-transfected with the pGLmP-539 *SerpinB2* promoter-luciferase reporter and β-galactosidase reporter plasmids, and luciferase units normalized to β-galactosidase activity. *(B)* C/EBP-β gene schematic depicting gene structure and phospho-acceptor sites. *(C)* Phosphorylation of C/EBP-β at Serine 64 negatively regulates LPS-stimulated *SerpinB2* promoter activity. *Cebpb^−/−^* MEFs were co-transfected with expression plasmids encoding wild-type C/EBP-β or the indicated C/EBP-β phospho-acceptor mutants, along with the pGLmP-539 *SerpinB2* promoter-luciferase reporter and β-galactosidase reporter plasmids, and stimulated with LPS for 4 hrs. Luciferase activity was quantified and normalized to β-galactosidase activity. The western blot (below the graphs) confirms the expression of the respective C/EBP-β phospho-acceptor mutants. The C/EBP-β^T217A^ -transfected MEFs express both the 38 kDa and 35 kDa isoforms of C/EBP-β. The results represent the mean and SEM of at least three independent experiments performed in triplicate. (*, p<0.05, one-way ANOVA).

Since C/EBP-β binds to the *SerpinB2* proximal promoter in an LPS-inducible manner both *in vitro* and *in vivo*, we wanted to address the question of whether C/EBP-β was an essential factor for driving transcription from the *SerpinB2* promoter in cells in response to LPS. The ability of endogenous C/EBP-β to direct transcription from the *SerpinB2* proximal promoter was examined by transfection of the pGLmP-539 murine *SerpinB2* luciferase reporter construct into *Cebpb^+/+^* and *Cebpb^−/−^* MEFs. We found that LPS-stimulated *SerpinB2* promoter activity was significantly increased in *Cebpb^+/+^* MEFs and abrogated in *Cebpb^−/−^* MEFs (Fig. 8A), indicating that endogenous C/EBP-β is required for LPS-induced *SerpinB2* proximal promoter activity.

### Phosphorylation of C/EBP-β at Serine 64 negatively Regulates LPS-stimulated SerpinB2 Promoter Activity

Phosphorylation of C/EBP-β is well recognized to modulate its transactivation potential [63]. To investigate C/EBP-β phosphorylation sites that may be important for LPS-stimulated *SerpinB2* proximal promoter activity (Fig. 8B), we co-expressed several C/EBP-β phospho-acceptor mutants in which the critical threonine or serine residue was mutated to an alanine, along with the pGLmP-539 murine *SerpinB2* luciferase reporter in *Cebpb^−/−^* MEFs. Re-expression of wild-type C/EBP-β in *Cebpb^−/−^* MEFs significantly stimulated *SerpinB2* luciferase reporter gene expression in the presence of LPS by ∼3 fold (Fig. 8C, left), confirming the importance of C/EBP-β to LPS-induced *SerpinB2* gene transcription. Expression of the C/EBP-β phospho-acceptor mutant, C/EBPβ^T217A^, in *Cebpb^−/−^* MEFs did not significantly increase *SerpinB2* promoter activity above that of wild-type C/EBP-β (Fig. 8C, right); confirming that phosphorylation of C/EBP-β at T^217^ is not a major factor in the regulation of *SerpinB2* promoter activity in response to LPS.

C/EBP-β contains additional phosphorylation sites, C/EBP-β^T188^ and C/EBP-β^S64^ (Fig. 8B), which may be involved in modulating C/EBP-β-dependent *SerpinB2* gene transcription. C/EBP-β^T188^ is implicated in regulating DAPK1, an IFNγ-inducible gene involved in the regulation of cell cycle and apoptosis [38], processes with which SerpinB2 has also been associated [17]. C/EBP-β^S64^ is important for LPS-induced transcription of the cytokines IL-6 and MCP-1 [64]. Similarly, SerpinB2 is induced by LPS and regulated in a manner similar to cytokines [29]. Given the similarities in the functional significance of these phospho-specific isoforms of C/EBP-β and SerpinB2, we investigated whether these phospho-acceptor sites may play a role in *SerpinB2* gene expression. C/EBP-β^T188A^-transfected MEFs exhibited *SerpinB2* promoter activity similar to that of wild-type C/EBP-β-transfected MEFs, whereas the expression of C/EBP-β^S64A^ potentiated *SerpinB2* promoter activity in response to LPS (Fig. 8C, right). These data suggest that phosphorylation of C/EBP-β at S^64^ acts to negatively regulate *SerpinB2* proximal promoter activity.

## Discussion

Macrophages are key mediators of the innate immune response, and consequently provide the first line of defense against pathogens. Pro-inflammatory stimuli, such as the bacterial endotoxin LPS, stimulate macrophages to mount an anti-pathogenic response which involves massive induction of the pro-survival factor SerpinB2. SerpinB2 is transcriptionally induced by cross talk between the IKKβ/NF-κB and p38MAPK signaling modules in response to LPS [16]. Here we report that SerpinB2 gene transcription in response to LPS is conferred by the SerpinB2 proximal promoter and is greatly dependent upon C/EBP-β. LPS-induced C/EBP-β was shown to specifically bind the C/EBP response element in the SerpinB2 proximal promoter in vitro and in vivo, and loss of C/EBP-β abrogates constitutive SerpinB2 gene transcription and the response to LPS.

The murine *SerpinB2* proximal promoter region between nucleotides -539 and +92 mediated both PMA- and LPS-inducible gene transcription, with induction by PMA being less intense and more transient than that by LPS. Inspection of the murine *SerpinB2* proximal promoter sequence shows that a CRE and two AP-1-like elements, demonstrated to mediate PMA-stimulated transcription of the human *SERPINB2* gene [37], also are present in the murine *SerpinB2* proximal promoter between nucleotides −189 and −87. These sites may therefore also play a role in mediating PMA-inducible transcription of the murine *SerpinB2* gene. In contrast to the pattern of incremental increases in PMA-induced transcriptional activity conferred by regions of the murine *SerpinB2* promoter containing these sites, most of the LPS-inducible response is dependent upon *cis*-acting regulatory sequences in the region between nucleotides −189 and −539. LPS responsiveness absolutely required the C/EBP binding site located in the region between nucleotides −189 and −539, with the downstream CRE and AP-1-like elements also being critical. Of note, there are previous reports of combinatorial interactions between C/EBP-β and CRE binding proteins (CREB) and AP-1 [63], and C/EBP-β has been reported to physically interact with AP-1, and NF-κB to promote gene expression of inflammatory mediators [63]; [66]. Additionally, CREB has been shown to control transcription of the C/EBP-β gene [67].

In this study, C/EBP-β was found to be a major requirement for both constitutive and LPS-induced *SerpinB2* gene transcription. In a previous microarray expression profiling study, *SerpinB2* was identified as gene whose induction in C/EBP-β-deficient peritoneal macrophages by LPS and IFNγ was severely impaired compared to wild-type macrophages [68]. Our qPCR results validate this finding, as we found that both constitutive and LPS-inducible *SerpinB2* mRNA expression is significantly abrogated in C/EBP-β-shRNA transduced peritoneal macrophages, emphasizing the link between C/EBP-β and *SerpinB2* gene transcription. While C/EBP proteins can act as either homodimers or heterodimers [63], we identified C/EBP-β as the only LPS-inducible C/EBP isoform to bind the *SerpinB2* C/EBP response element, suggesting a predominant role for C/EBP-β in LPS-induced *SerpinB2* gene expression. C/EBP-β, like SerpinB2, plays an important role in inflammation, as it is upregulated by LPS and a host of other inflammatory cytokines [59]; [62]. Furthermore, C/EBP-β-null mice are susceptible to bacterial infection [69]; [70] and SerpinB2 has been demonstrated to protect from bacterial and viral-induced cell death [14]–[16]; [25]. Thus the regulation of SerpinB2 gene expression by C/EBP-β is consistent with its functional role in inflammation.

Phosphorylation of C/EBP-β at several different amino acid residues has been shown to modulate transactivation of its target genes [63]; . C/EBP-β phosphorylated on T^217^ has been reported to rescue macrophages from apoptosis induced by *Bacillus anthracis* lethal toxin (LT) [65], an activity that has also been attributed to SerpinB2 [16]. However, expression of a C/EBP-β phospho-acceptor site mutant, C/EBPβ^T217A^, in *Cebpb^−/−^* MEFs did not increase *SerpinB2* proximal promoter activity over that of wild-type C/EBP-β, even though recruitment of C/EBP-β^T217^ to the *SerpinB2* promoter was observed to decrease following LPS stimulation of RAW264.7 cells. These data indicate that the T^217^ phospho-acceptor site is not important for the regulation of *SerpinB2* gene expression. Similarly, expression of the C/EBP-β phospho-acceptor site mutant, C/EBP-β^T188A^, did not affect *SerpinB2* promoter activity differently from that of wild-type. In contrast, expression of C/EBP-β^S64A^ significantly enhanced LPS-induced *SerpinB2* promoter activity, indicating that phosphorylation of C/EBP-β at S^64^ negatively regulates LPS-induced *SerpinB2* promoter activity. Since C/EBP-β S^64^ is constitutively phosphorylated in both RAW264.7 cells and MEFs [73], it is likely that dephosphorylation at this site may be a critical event during LPS-induced transcription of *SerpinB2* to increase its promoter activity. Roy and colleagues demonstrated that Mixed lineage kinase-3 (MLK3)-driven dephosphorylation of C/EBP-β S^64^ was important for IFNγ-regulated signaling pathways [73]. Our data suggest that MLK3-driven dephosphorylation of S^64^ may also be involved in LPS-signaling pathways.

In recent years it has become apparent that persistent infection is integrally linked to chronic inflammation and cancer, and immune cells such as macrophages can either promote or attenuate cancer progression [2]; [74]–[76]. SerpinB2 expression has been associated with both inflammation and cancer, and is a favorable or unfavorable prognostic indicator depending on cancer type [77]. The presence of SerpinB2 has been shown to modulate cytokine profiles which can affect immune cell polarization [27]; [28]; [77]; [78]. Interestingly C/EBP-β has also been shown to modulate cytokine secretion from immune cells, thereby modifying their phenotype [27]; [79]–[81]. Our study has demonstrated that C/EBP-β plays an important role in mediating both constitutive and LPS-induced transcription of the *SerpinB2* gene, which may have implications for the inflammatory phenotype of infiltrating immune cells in the tumor microenvironment.

In summary, our studies show that the C/EBP site (−203/−195) in the murine *SerpinB2* proximal promoter is necessary to support both constitutive and LPS-induced *SerpinB2* gene expression. Importantly, we were able to uncover a previously unknown role for C/EBP-β^S64^ in negatively regulating SerpinB2 promoter activity. Taken together these data provide new insight into the regulation of inflammation-associated *SerpinB2* gene expression.

## Supporting Information

Figure S1 **PCR primer sequences used to generate murine SerpinB2 reporter constructs.** *(top)* PCR primer sequences used to subclone *SerpinB2* promoter sequences into pGL3 Basic luciferase reporter plasmid and generate deletion constructs as indicated in Experimental Procedures. KpnI (GGTACC) and XhoI (CTCGAG) restriction sites are underlined. *(bottom)* mutant oligonucleotide PCR primers used to mutate LPS-responsive regions in the SerpinB2 proximal promoter. Mutated nucleotides are shown in bold.
(TIF)

Figure S2 **Conserved response elements in the murine and human** ***SerpinB2*** **promoter.** Location and nucleotide sequences of the *cis*-acting elements conserved between the human and murine *SerpinB2* promoters are listed as indicated.
(TIF)

## References

[1] Cohen J (2002) The immunopathogenesis of sepsis. Nature 420: 885–891. 12490963 10.1038/nature01326

[2] Grivennikov SI, Greten FR, Karin M (2010) Immunity, inflammation, and cancer. Cell 140: 883–899. 20303878 10.1016/j.cell.2010.01.025 PMC2866629

[3] Grivennikov SI, Karin M (2010) Inflammation and oncogenesis: a vicious connection. Curr.Opin.Genet.Dev. 20: 65–71. 10.1016/j.gde.2009.11.004 PMC2821983 20036794

[4] Costelloe EO, Stacey KJ, Antalis TM, Hume DA (1999) Regulation of the plasminogen activator inhibitor-2 (PAI-2) gene in murine macrophages. Demonstration of a novel pattern of responsiveness to bacterial endotoxin. J.Leukoc.Biol. 66: 172–182. 10.1002/jlb.66.1.172 10411006

[5] Schwartz BS, Bradshaw JD (1992) Regulation of plasminogen activator inhibitor mRNA levels in lipopolysaccharide-stimulated human monocytes. Correlation with production of the protein. J.Biol.Chem. 267: 7089–7094. 1551915

[6] Antalis TM, Clark MA, Barnes T, Lehrbach PR, Devine PL, et al (1988) Cloning and expression of a cDNA coding for a human monocyte-derived plasminogen activator inhibitor. Proc.Natl.Acad.Sci.U.S.A 85: 985–989. 3257578 10.1073/pnas.85.4.985 PMC279685

[7] Vassalli JD, Sappino AP, Belin D (1991) The plasminogen activator/plasmin system. J.Clin.Invest 88: 1067–1072. 1833420 10.1172/JCI115405 PMC295552

[8] Webb AC, Collins KL, Snyder SE, Alexander SJ, Rosenwasser LJ, et al (1987) Human monocyte Arg-Serpin cDNA. Sequence, chromosomal assignment, and homology to plasminogen activator-inhibitor. J.Exp.Med. 166: 77–94. 10.1084/jem.166.1.77 PMC2188630 3496414

[9] Kruithof EK, Baker MS, Bunn CL (1995) Biological and clinical aspects of plasminogen activator inhibitor type 2. Blood 86: 4007–4024. 7492756

[10] Bird CH, Blink EJ, Hirst CE, Buzza MS, Steele PM, et al (2001) Nucleocytoplasmic distribution of the ovalbumin serpin PI-9 requires a nonconventional nuclear import pathway and the export factor Crm1. Mol.Cell Biol. 21: 5396–5407. 10.1128/MCB.21.16.5396-5407.2001 PMC87262 11463822

[11] Kumar S, Baglioni C (1991) Protection from tumor necrosis factor-mediated cytolysis by overexpression of plasminogen activator inhibitor type-2. J.Biol.Chem. 266: 20960–20964. 1939146

[12] Dickinson JL, Bates EJ, Ferrante A, Antalis TM (1995) Plasminogen activator inhibitor type 2 inhibits tumor necrosis factor alpha-induced apoptosis. Evidence for an alternate biological function. J.Biol.Chem. 270: 27894–27904. 10.1074/jbc.270.46.27894 7499264

[13] Dickinson JL, Norris BJ, Jensen PH, Antalis TM (1998) The C-D interhelical domain of the serpin plasminogen activator inhibitor-type 2 is required for protection from TNF-alpha induced apoptosis. Cell Death.Differ. 5: 163–171. 10.1038/sj.cdd.4400324 10200461

[14] Gan H, Newman GW, Remold HG (1995) Plasminogen activator inhibitor type 2 prevents programmed cell death of human macrophages infected with Mycobacterium avium, serovar 4. J.Immunol. 155: 1304–1315. 7636197

[15] Antalis TM, La Linn M, Donnan K, Mateo L, Gardner J, et al (1998) The serine proteinase inhibitor (serpin) plasminogen activation inhibitor type 2 protects against viral cytopathic effects by constitutive interferon alpha/beta priming. J.Exp.Med. 187: 1799–1811. 10.1084/jem.187.11.1799 PMC2212304 9607921

[16] Park JM, Greten FR, Wong A, Westrick RJ, Arthur JS, et al (2005) Signaling pathways and genes that inhibit pathogen-induced macrophage apoptosis–CREB and NF-kappaB as key regulators. Immunity. 23: 319–329. 10.1016/j.immuni.2005.08.010 16169504

[17] Bird PI (1998) Serpins and regulation of cell death. Results Probl.Cell Differ. 24: 63–89. 10.1007/978-3-540-69185-3_4 9949832

[18] Hibino T, Izaki S, Ohkuma M, Kon S, Thorsen S, et al (1988) Epidermal plasminogen activator inhibitor (PAI) is immunologically identical to placental-type PAI-2. FEBS Lett. 231: 202–206. 10.1016/0014-5793(88)80731-2 3129308

[19] Yu H, Maurer F, Medcalf RL (2002) Plasminogen activator inhibitor type 2: a regulator of monocyte proliferation and differentiation. Blood 99: 2810–2818. 11929770 10.1182/blood.v99.8.2810

[20] Lian X, Yang T (2004) Plasminogen activator inhibitor 2: expression and role in differentiation of epidermal keratinocyte. Biol.Cell 96: 109–116. 15050365 10.1016/j.biolcel.2003.09.007

[21] Jensen PJ, Lavker RM (1996) Modulation of the plasminogen activator cascade during enhanced epidermal proliferation in vivo. Cell Growth Differ. 7: 1793–1804. 8959348

[22] Shafren DR, Gardner J, Mann VH, Antalis TM, Suhrbier A (1999) Picornavirus receptor down-regulation by plasminogen activator inhibitor type 2. J.Virol. 73: 7193–7198. 10.1128/jvi.73.9.7193-7198.1999 PMC104243 10438806

[23] Darnell GA, Antalis TM, Johnstone RW, Stringer BW, Ogbourne SM, et al (2003) Inhibition of retinoblastoma protein degradation by interaction with the serpin plasminogen activator inhibitor 2 via a novel consensus motif. Mol.Cell Biol. 23: 6520–6532. 10.1128/MCB.23.18.6520-6532.2003 PMC193706 12944478

[24] Varro A, Noble PJ, Pritchard DM, Kennedy S, Hart CA, et al (2004) Helicobacter pylori induces plasminogen activator inhibitor 2 in gastric epithelial cells through nuclear factor-kappaB and RhoA: implications for invasion and apoptosis. Cancer Res. 64: 1695–1702. 10.1158/0008-5472.can-03-2399 14996729

[25] Gan H, Lee J, Ren F, Chen M, Kornfeld H, et al (2008) Mycobacterium tuberculosis blocks crosslinking of annexin-1 and apoptotic envelope formation on infected macrophages to maintain virulence. Nat.Immunol. 9: 1189–1197. 10.1038/ni.1654 PMC5351782 18794848

[26] Losick VP, Isberg RR (2006) NF-kappaB translocation prevents host cell death after low-dose challenge by Legionella pneumophila. J Exp.Med. 203: 2177–2189. 10.1084/jem.20060766 PMC2118400 16940169

[27] Schroder WA, Gardner J, Le TT, Duke M, Burke ML, et al (2010) SerpinB2 deficiency modulates Th1Th2 responses after schistosome infection. Parasite Immunol. 32: 764–768. 10.1111/j.1365-3024.2010.01241.x 21086717

[28] Schroder WA, Le TT, Major L, Street S, Gardner J, et al (2010) A physiological function of inflammation-associated SerpinB2 is regulation of adaptive immunity. J.Immunol. 184: 2663–2670. 10.4049/jimmunol.0902187 20130210

[29] Stasinopoulos S, Mariasegaram M, Gafforini C, Nagamine Y, Medcalf RL (2010) The plasminogen activator inhibitor 2 transcript is destabilized via a multi-component 3' UTR localized adenylate and uridylate-rich instability element in an analogous manner to cytokines and oncogenes. FEBS J. 277: 1331–1344. 10.1111/j.1742-4658.2010.07563.x 20392207

[30] Antalis TM, Costelloe E, Muddiman J, Ogbourne S, Donnan K (1996) Regulation of the plasminogen activator inhibitor type-2 gene in monocytes: localization of an upstream transcriptional silencer. Blood 88: 3686–3697. 8916932

[31] Medcalf RL (2011) Plasminogen activator inhibitor type 2: still an enigmatic serpin but a model for gene regulation. Methods Enzymol. 499: 105–134. 10.1016/B978-0-12-386471-0.00006-7 21683251

[32] Suzuki T, Hashimoto S, Toyoda N, Nagai S, Yamazaki N, et al (2000) Comprehensive gene expression profile of LPS-stimulated human monocytes by SAGE. Blood 96: 2584–2591. 11001915

[33] Akira S, Takeda K (2004) Toll-like receptor signalling. Nat.Rev.Immunol. 4: 499–511. 10.1038/nri1391 15229469

[34] Roy SK, Hu J, Meng Q, Xia Y, Shapiro PS, et al (2002) MEKK1 plays a critical role in activating the transcription factor C/EBP-beta-dependent gene expression in response to IFN-gamma. Proc.Natl.Acad.Sci.U.S.A 99: 7945–7950. 12048245 10.1073/pnas.122075799 PMC123000

[35] Dougherty KM, Pearson JM, Yang AY, Westrick RJ, Baker MS, et al (1999) The plasminogen activator inhibitor-2 gene is not required for normal murine development or survival. Proc.Natl.Acad.Sci.U.S.A 96: 686–691. 9892694 10.1073/pnas.96.2.686 PMC15197

[36] Ho SN, Hunt HD, Horton RM, Pullen JK, Pease LR (1989) Site-directed mutagenesis by overlap extension using the polymerase chain reaction. Gene 77: 51–59. 2744487 10.1016/0378-1119(89)90358-2

[37] Cousin E, Medcalf RL, Bergonzelli GE, Kruithof EK (1991) Regulatory elements involved in constitutive and phorbol ester-inducible expression of the plasminogen activator inhibitor type 2 gene promoter. Nucleic Acids Res. 19: 3881–3886. 10.1093/nar/19.14.3881 PMC328478 1650454

[38] Gade P, Roy SK, Li H, Nallar SC, Kalvakolanu DV (2008) Critical role for transcription factor C/EBP-beta in regulating the expression of death-associated protein kinase 1. Mol.Cell Biol. 28: 2528–2548. 10.1128/MCB.00784-07 PMC2293111 18250155

[39] Buck M, Poli V, van der Geer P, Chojkier M, Hunter T (1999) Phosphorylation of rat serine 105 or mouse threonine 217 in C/EBP beta is required for hepatocyte proliferation induced by TGF alpha. Mol.Cell 4: 1087–1092. 10635333 10.1016/s1097-2765(00)80237-3

[40] Shuman JD, Sebastian T, Kaldis P, Copeland TD, Zhu S, et al (2004) Cell cycle-dependent phosphorylation of C/EBPbeta mediates oncogenic cooperativity between C/EBPbeta and H-RasV12. Mol.Cell Biol. 24: 7380–7391. 10.1128/MCB.24.17.7380-7391.2004 PMC507001 15314150

[41] Baer M, Johnson PF (2000) Generation of truncated C/EBPbeta isoforms by in vitro proteolysis. J.Biol.Chem. 275: 26582–26590. 10.1074/jbc.M004268200 10856306

[42] Chang S, Stacey KJ, Chen J, Costelloe EO, Aderem A, et al (1999) Mechanisms of regulation of the MacMARCKS gene in macrophages by bacterial lipopolysaccharide. J.Leukoc.Biol. 66: 528–534. 10.1002/jlb.66.3.528 10496325

[43] Maurer F, Tierney M, Medcalf RL (1999) An AU-rich sequence in the 3'-UTR of plasminogen activator inhibitor type 2 (PAI-2) mRNA promotes PAI-2 mRNA decay and provides a binding site for nuclear HuR. Nucleic Acids Res. 27: 1664–1673. 10.1093/nar/27.7.1664 PMC148370 10075998

[44] Tierney MJ, Medcalf RL (2001) Plasminogen activator inhibitor type 2 contains mRNA instability elements within exon 4 of the coding region. Sequence homology to coding region instability determinants in other mRNAs. J.Biol.Chem. 276: 13675–13684. 10.1074/jbc.M010627200 11278713

[45] Bergonzelli GE, Kruithof EK, Medcalf RL (1992) Transcriptional antagonism of phorbol ester-mediated induction of plasminogen activator inhibitor types 1 and 2 by cyclic adenosine 3',5'-monophosphate. Endocrinology 131: 1467–1472. 1354603 10.1210/endo.131.3.1354603

[46] Xiao G, Liu YE, Gentz R, Sang QA, Ni J, et al (1999) Suppression of breast cancer growth and metastasis by a serpin myoepithelium-derived serine proteinase inhibitor expressed in the mammary myoepithelial cells. Proc.Natl.Acad.Sci.U.S.A 96: 3700–3705. 10097100 10.1073/pnas.96.7.3700 PMC22357

[47] Stringer B, Udofa EA, Antalis TM (2012) Regulation of the human plasminogen activator inhibitor type 2 (PAI-2) gene: cooperation of an upstream silencer and transactivator. J.Biol.Chem. 10.1074/jbc.M111.318758 PMC3322994 22334683

[48] Schleuning WD, Medcalf RL, Hession C, Rothenbuhler R, Shaw A, et al (1987) Plasminogen activator inhibitor 2: regulation of gene transcription during phorbol ester-mediated differentiation of U-937 human histiocytic lymphoma cells. Mol.Cell Biol. 7: 4564–4567. 10.1128/mcb.7.12.4564-4567.1987 PMC368144 3325828

[49] Maurer F, Medcalf RL (1996) Plasminogen activator inhibitor type 2 gene induction by tumor necrosis factor and phorbol ester involves transcriptional and post-transcriptional events. Identification of a functional nonameric AU-rich motif in the 3'-untranslated region. J Biol.Chem. 271: 26074–26080. 10.1074/jbc.271.42.26074 8824249

[50] Antalis TM, Dickinson JL (1992) Control of plasminogen-activator inhibitor type 2 gene expression in the differentiation of monocytic cells. Eur.J.Biochem. 205: 203–209. 10.1111/j.1432-1033.1992.tb16769.x 1555580

[51] Antalis TM, Godbolt D, Donnan KD, Stringer BW (1993) Southwestern blot mapping of potential regulatory proteins binding to the DNA encoding plasminogen activator inhibitor type 2. Gene 134: 201–208. 8262378 10.1016/0378-1119(93)90094-j

[52] Hardison RC (2000) Conserved noncoding sequences are reliable guides to regulatory elements. Trends Genet. 16: 369–372. 10.1016/s0168-9525(00)02081-3 10973062

[53] Ye RD, Wun TC, Sadler JE (1987) cDNA cloning and expression in Escherichia coli of a plasminogen activator inhibitor from human placenta. J.Biol.Chem. 262: 3718–3725. 3029122

[54] Samia JA, Alexander SJ, Horton KW, Auron PE, Byers MG, et al (1990) Chromosomal organization and localization of the human urokinase inhibitor gene: perfect structural conservation with ovalbumin. Genomics 6: 159–67. 2303256 10.1016/0888-7543(90)90461-3

[55] Dear AE, Costa M, Medcalf RL (1997) Urokinase-mediated transactivation of the plasminogen activator inhibitor type 2 (PAI-2) gene promoter in HT-1080 cells utilises AP-1 binding sites and potentiates phorbol ester-mediated induction of endogenous PAI-2 mRNA. FEBS Lett. 402: 265–272. 10.1016/s0014-5793(97)00002-1 9037208

[56] Schuster WA, Medcalf RL, Kruithof EKO (1994) Localization and Characterization of a Retinoic Acid Response-Like Element in the Plasminogen Activator Inhibitor-2 Gene Promoter. Fibrinolysis 8: 113–9.

[57] Sweet MJ, Hume DA (1996) Endotoxin signal transduction in macrophages. J.Leukoc.Biol. 60: 8–26. 10.1002/jlb.60.1.8 8699127

[58] Sekine H, Mimura J, Oshima M, Okawa H, Kanno J, et al (2009) Hypersensitivity of aryl hydrocarbon receptor-deficient mice to lipopolysaccharide-induced septic shock. Mol.Cell Biol. 29: 6391–6400. 10.1128/MCB.00337-09 PMC2786870 19822660

[59] Poli V (1998) The role of C/EBP isoforms in the control of inflammatory and native immunity functions. J.Biol.Chem. 273: 29279–29282. 10.1074/jbc.273.45.29279 9792624

[60] Kalvakolanu DV, Roy SK (2005) CCAAT/enhancer binding proteins and interferon signaling pathways. J.Interferon Cytokine Res. 25: 757–769. 10.1089/jir.2005.25.757 16375604

[61] Hirai H, Zhang P, Dayaram T, Hetherington CJ, Mizuno S, et al (2006) C/EBPbeta is required for 'emergency' granulopoiesis. Nat.Immunol. 7: 732–739. 10.1038/ni1354 16751774

[62] Bradley MN, Zhou L, Smale ST (2003) C/EBPbeta regulation in lipopolysaccharide-stimulated macrophages. Mol.Cell Biol. 23: 4841–4858. 10.1128/MCB.23.14.4841-4858.2003 PMC162211 12832471

[63] Tsukada J, Yoshida Y, Kominato Y, Auron PE (2011) The CCAAT/enhancer (C/EBP) family of basic-leucine zipper (bZIP) transcription factors is a multifaceted highly-regulated system for gene regulation. Cytokine 54: 6–19. 21257317 10.1016/j.cyto.2010.12.019

[64] Spooner CJ, Sebastian T, Shuman JD, Durairaj S, Guo X, et al (2007) C/EBPbeta serine 64, a phosphoacceptor site, has a critical role in LPS-induced IL-6 and MCP-1 transcription. Cytokine 37: 119–127. 17433708 10.1016/j.cyto.2007.03.001

[65] Buck M, Chojkier M (2007) C/EBPbeta phosphorylation rescues macrophage dysfunction and apoptosis induced by anthrax lethal toxin. Am.J.Physiol Cell Physiol 293: C1788–C1796. 17855774 10.1152/ajpcell.00141.2007

[66] Matsusaka T, Fujikawa K, Nishio Y, Mukaida N, Matsushima K, et al (1993) Transcription factors NF-IL6 and NF-kappa B synergistically activate transcription of the inflammatory cytokines, interleukin 6 and interleukin 8. Proc.Natl.Acad.Sci.U.S.A 90: 10193–10197. 8234276 10.1073/pnas.90.21.10193 PMC47740

[67] Niehof M, Manns MP, Trautwein C (1997) CREB controls LAP/C/EBP beta transcription. Mol.Cell Biol. 17: 3600–3613. 10.1128/mcb.17.7.3600 PMC232213 9199295

[68] Uematsu S, Kaisho T, Tanaka T, Matsumoto M, Yamakami M, et al (2007) The C/EBP beta isoform 34-kDa LAP is responsible for NF-IL-6-mediated gene induction in activated macrophages, but is not essential for intracellular bacteria killing. J.Immunol. 179: 5378–5386. 10.4049/jimmunol.179.8.5378 17911624

[69] Tanaka T, Akira S, Yoshida K, Umemoto M, Yoneda Y, et al (1995) Targeted disruption of the NF-IL6 gene discloses its essential role in bacteria killing and tumor cytotoxicity by macrophages. Cell 80: 353–361. 7530603 10.1016/0092-8674(95)90418-2

[70] Lekstrom-Himes J, Xanthopoulos KG (1998) Biological role of the CCAAT/enhancer-binding protein family of transcription factors. J.Biol.Chem. 273: 28545–28548. 10.1074/jbc.273.44.28545 9786841

[71] Ramji DP, Foka P (2002) CCAAT/enhancer-binding proteins: structure, function and regulation. Biochem.J. 365: 561–575. 10.1042/BJ20020508 PMC1222736 12006103

[72] Schrem H, Klempnauer J, Borlak J (2004) Liver-enriched transcription factors in liver function and development. Part II: the C/EBPs and D site-binding protein in cell cycle control, carcinogenesis, circadian gene regulation, liver regeneration, apoptosis, and liver-specific gene regulation. Pharmacol.Rev. 56: 291–330. 10.1124/pr.56.2.5 15169930

[73] Roy SK, Shuman JD, Platanias LC, Shapiro PS, Reddy SP, et al (2005) A role for mixed lineage kinases in regulating transcription factor CCAAT/enhancer-binding protein-{beta}-dependent gene expression in response to interferon-{gamma}. J.Biol.Chem. 280: 24462–24471. 10.1074/jbc.M413661200 15878863

[74] Karin M, Lawrence T, Nizet V (2006) Innate immunity gone awry: linking microbial infections to chronic inflammation and cancer. Cell 124: 823–835. 16497591 10.1016/j.cell.2006.02.016

[75] Karin M, Greten FR (2005) NF-kappaB: linking inflammation and immunity to cancer development and progression. Nat.Rev.Immunol. 5: 749–759. 10.1038/nri1703 16175180

[76] DiDonato JA, Mercurio F, Karin M (2012) NF-kappaB and the link between inflammation and cancer. Immunol.Rev. 246: 379–400. 10.1111/j.1600-065X.2012.01099.x 22435567

[77] Croucher DR, Saunders DN, Lobov S, Ranson M (2008) Revisiting the biological roles of PAI2 (SERPINB2) in cancer. Nat.Rev.Cancer 8: 535–545. 18548086 10.1038/nrc2400

[78] Schroder WA, Major L, Suhrbier A (2011) The role of SerpinB2 in immunity. Crit Rev.Immunol. 31: 15–30. 10.1615/critrevimmunol.v31.i1.20 21395508

[79] Greten FR, Arkan MC, Bollrath J, Hsu LC, Goode J, et al (2007) NF-kappaB is a negative regulator of IL-1beta secretion as revealed by genetic and pharmacological inhibition of IKKbeta. Cell 130: 918–931. 17803913 10.1016/j.cell.2007.07.009 PMC2134986

[80] Marigo I, Bosio E, Solito S, Mesa C, Fernandez A, et al (2010) Tumor-induced tolerance and immune suppression depend on the C/EBPbeta transcription factor. Immunity. 32: 790–802. 10.1016/j.immuni.2010.05.010 20605485

[81] Screpanti I, Romani L, Musiani P, Modesti A, Fattori E, et al (1995) Lymphoproliferative disorder and imbalanced T-helper response in C/EBP beta-deficient mice. EMBO J. 14: 1932–1941. 10.1002/j.1460-2075.1995.tb07185.x PMC398292 7744000

