# Supplementary figures and images for: The Transcription Factor C/EBP-β Mediates Constitutive and LPS-Inducible Transcription of Murine SerpinB2
Source: PLoS One. 2013 Mar 5;8(3):e57855. doi: 10.1371/journal.pone.0057855 (PMC3589482; doi:10.1371/journal.pone.0057855)

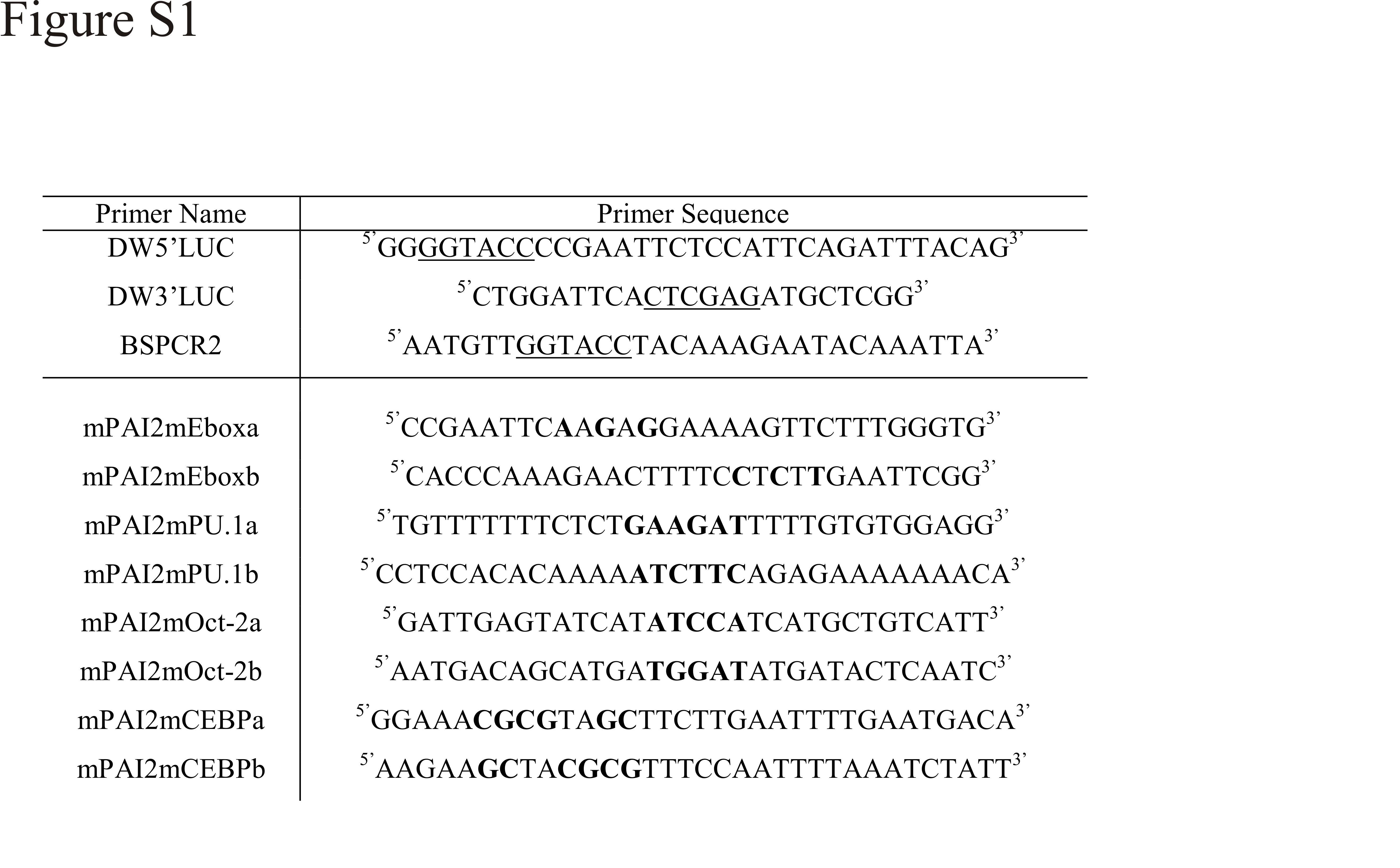

Supplement: Figure S1 — PCR primer sequences used to generate murine SerpinB2 reporter constructs. (top) PCR primer sequences used to subclone SerpinB2 promoter sequences into pGL3 Basic luciferase reporter plasmid and generate deletion constructs as indicated in Experimental Procedures. KpnI (GGTACC) and XhoI (CTCGAG) restriction sites are underlined. (bottom) mutant oligonucleotide PCR primers used to mutate LPS-responsive regions in the SerpinB2 proximal promoter. Mutated nucleotides are shown in bold. (TIF) [file pone.0057855.s001.tif]

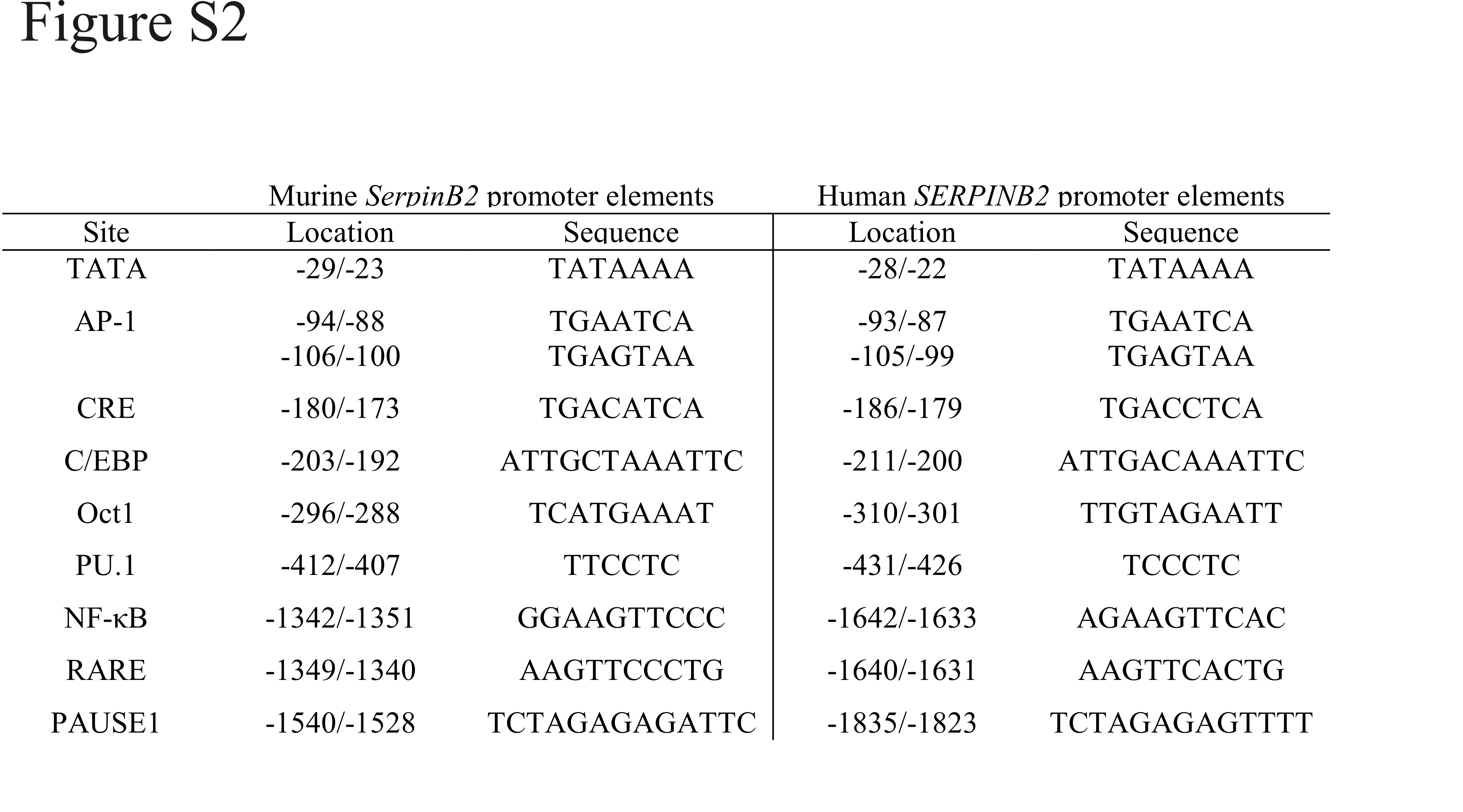

Supplement: Figure S2 — Conserved response elements in the murine and human SerpinB2 promoter. Location and nucleotide sequences of the cis-acting elements conserved between the human and murine SerpinB2 promoters are listed as indicated. (TIF) [file pone.0057855.s002.tif]
